# Network analysis of the Viking Age in Ireland as portrayed in *Cogadh Gaedhel re Gallaibh*

**DOI:** 10.1098/rsos.171024

**Published:** 2018-01-24

**Authors:** Joseph Yose, Ralph Kenna, Máirín MacCarron, Pádraig MacCarron

**Affiliations:** 1 Applied Mathematics Research Centre, Coventry University, Coventry CV1 5FB, UK; 2 Department of History, University of Sheffield, Sheffield S3 7RA, UK; 3 Social and Evolutionary Neuroscience Research Group, Department of Experimental Psychology, University of Oxford, Oxford OX1 3UD, UK

**Keywords:** character networks, assortativity, epic narratives, Vikings, Ireland, complexity science

## Abstract

*Cogadh Gaedhel re Gallaibh* (‘The War of the Gaedhil with the Gaill’) is a medieval Irish text, telling how an army under the leadership of Brian Boru challenged Viking invaders and their allies in Ireland, culminating with the Battle of Clontarf in 1014. Brian’s victory is widely remembered for breaking Viking power in Ireland, although much modern scholarship disputes traditional perceptions. Instead of an international conflict between Irish and Viking, interpretations based on revisionist scholarship consider it a domestic feud or civil war. Counter-revisionists challenge this view and a long-standing and lively debate continues. Here, we introduce quantitative measures to the discussions. We present statistical analyses of network data embedded in the text to position its sets of interactions on a spectrum from the domestic to the international. This delivers a picture that lies between antipodal traditional and revisionist extremes; hostilities recorded in the text are mostly between Irish and Viking—but internal conflict forms a significant proportion of the negative interactions too.

## 1. Introduction

Modern academic disciplines do not exist in isolation and are increasingly interdependent and interconnected. For example, our understanding of the past utilizes scientific analyses of archaeological data, anthropology derives from evolutionary biology and economics requires mathematics and statistics. Statistical physics-inspired methodologies have long been applied to other academic disciplines, motivated not least by curiosity as to how complex systems emerge from interactions between constituent parts in non-trivial manners. Scientific curiosity of this kind has led to the development of new interdisciplinary areas and the creation of new knowledge by thinking beyond traditional methodological boundaries. In recent years, facilitated by new access to extensive datasets and technological progress, many statistical physicists have broadened their interests to include network science, a methodology which has led to an explosion of interdisciplinary activity. While many social-network studies focus on modern forms of sociality such as online communications and other forms of computer-mediated social media, the importance of exploring other kinds of data is increasingly recognized as well. In particular, quantitative investigations of epic narratives can advance our understanding of the past. A plethora of quantitative approaches and suggestions to investigate societal and cultural aspects of the past are contained in the compendium [1]. Here, we apply and develop one such method to a long-standing debate about the Viking Age in Ireland.

The Battle of Clontarf (1014), an iconic event in the history of Ireland, is traditionally remembered as marking the decline of Viking power after some two centuries in the country. For the past 250 years, a debate has been taking place centred around what may be called ‘traditionalist’ and ‘revisionist’ views of the period [2–7]. The recent millennial anniversary of the battle inspired academics to revisit the debate through new journal papers, books, booklets, monographs, online commentaries and media engagements (e.g. [7–18]). As with earlier investigations, these approaches treat the subject matter using traditional tools of the humanities (e.g. [19–48]). Here, we present an alternative, complexity science-based investigation, using one of the most famous accounts of the Vikings in Ireland: *Cogadh Gaedhel re Gallaibh*^1^ (‘The war of the Gaedhil with the Gaill’ or ‘War of the Irish with the Foreigners’).

The Viking Age in Ireland approximately spans the ninth to twelfth centuries. The *Cogadh* starts with the arrival of the Vikings^2^ (in 795) and gives a chronicle of their various raids. This is followed by a discussion of the Irish Dál Cais dynasty, their deeds, and those of their leader, Brian Boru, culminating in the Battle of Clontarf in 1014. Although its limitations are well documented, the text provides extensive information; it tells of multitudes of characters, alliances, conflicts, relationships and interactions of all sorts, from a perspective of when it was written. Statistical tools to tackle the networks formed by such large casts of characters have recently been developed [49–51]. Here, we apply them in a new investigation to shed quantitative light on the Viking Age in Ireland as presented in *Cogadh Gaedhel re Gallaibh*.

Network science is a broad academic field, related to statistical physics, information visualization, mathematical sociology and other disciplines [52–55]. It enables statistical treatment of certain types of systems comprising large numbers of interdependent elements. In character networks, these elements are individual figures (personages), represented by nodes (or vertices), and the interactions or relationships between them are represented by edges (or links). Empirical approaches seek to capture statistics which characterize such systems [55]. Besides delivering new quantitative insights when applied to old problems, the networks approach inspires new questions and opens new avenues of research.

The events associated with the Viking Age in Ireland and Battle of Clontarf are nowadays frequently considered as having entered the public imagination in an overly simplified manner. That popular picture is essentially of an ‘international’ conflict—Irish versus Viking—in which victory for the former ended the latter’s ambitions in the country.^3^ The truth, we are told, is more nuanced and more complex [5,6]. Instead of an international conflict, the issue at stake at Clontarf was an internal, domestic, Irish struggle: the determination of Leinster (in the east of Ireland) to remain independent of the dominant dynasties to its north and south-west [5,6]. Some such interpretations, wherein the Vikings are said to have played a secondary role, tend to downplay the significance of Clontarf [16] and have been partly ascribed to revisionist fashions [36,7]. *Cogadh Gaedhel re Gallaibh* has been used to bolster arguments on both sides of the debate. Our aim is to determine what its character networks have to say on the matter.

It is important to state from the outset that our analysis is of the content of *Cogadh Gaedhel re Gallaibh* and its portrayal of the Viking Age in Ireland. We do not have direct access to the actual social networks of the period and we recognize that the account in the *Cogadh* has been influenced by events and circumstances after 1014 and up to the composition of the text. We discuss the authenticity and deficiencies of the *Cogadh* as a source in §2.2. Nevertheless, the text is important in its own right and, at minimum, tells us how the author sought to represent reality.

The style of the text of the *Cogadh* is ‘inflated and bombastic’ [4]. It is considered by modern scholars ‘as a piece of dynastic political propaganda on behalf of the principal lineage of the Dál Cais, the Uí Briain’^4^ [27]. (See appendix A and figure 3 for a brief account of the political structure of Ireland in 1014.) This is achieved through extensive and elaborate passages extolling the virtues of Brian and his army while condemning the Vikings as brutal and piratical. However, such qualitative, rhetorical features are largely irrelevant for quantitative character-network analysis. Instead, our approach draws only from the most basic information—the presence or absence of interactions between characters. If the text contains networks which are reasonably or approximately reliable in the aggregate, they deliver useful information on the society of the time it presents.

**Figure 1.**
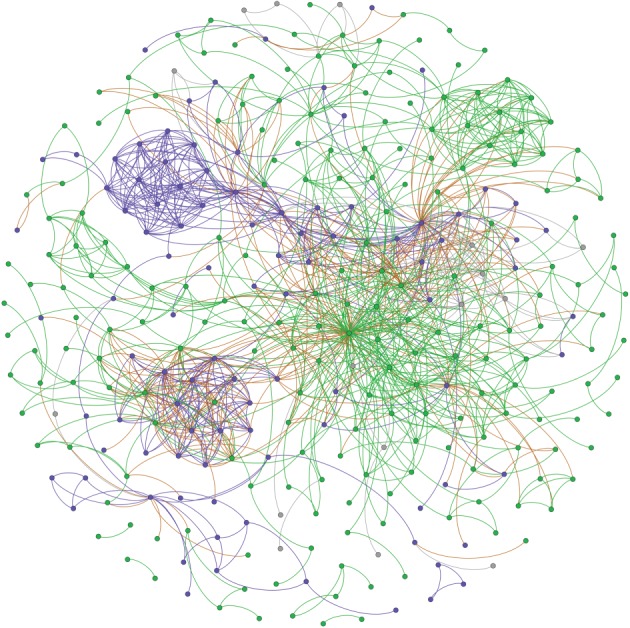
The entire *Cogadh* network of interacting characters. Characters identified as Irish are represented by green nodes and those identified as Vikings are in blue. Other characters are in grey. Edges between pairs of Irish nodes are also coloured green while those between Viking pairs are blue. Edges linking Irish to Viking nodes are brown and the remaining edges are grey.

The entire set of interacting characters in *Cogadh Gaedhel re Gallaibh* and the relationships between them is represented in figure 1 of §3. The figure represents a network of considerable complexity, similar to those of other epic narratives [49–51]. We are interested in the question whether the *Cogadh* networks are consistent with the traditional depiction of a contest which is clear-cut international or if they support the revisionist notion of a power-struggle which is mostly domestic or, indeed, if they deliver something between both pictures. A simple tally of edges (interactions between characters) will not do as this would not account for different numbers of Irish and Viking nodes, and a proper quantitative approach instead necessitates the networks-science concepts of *assortativity* and *disassortativity*. The former is the tendency for edges to connect nodes which have similar attributes. The opposite tendency is disassortativity; whereby links tend to be between nodes of different types. The type of attribute we are interested in here is *narrative identity*^5^ —categorized as Irish, Viking or other, and taken from the text itself. We wish to gauge whether nodes linked by different types of edges represent Irish or Viking characters as presented in the narrative. We use the generic term *categorical assortativity* for associated measures which will be used as the primary determinator to distinguish between the alternatives listed. A network with a positive value is said to be *categorically assortative*. A negative value signals disassortativity and a value close to zero indicates the absence of any such correlations (neither assortative nor disassortative).

We will report that the categorical assortativity for the conflictual network is moderately negative. This statistical approach suggests that while the *Cogadh* account is not as clear cut as either the most traditional or revisionist pictures in the debate depict, it lies on the traditional side. Thus, the networks of *Cogadh Gaedhel re Gallaibh* give a complex picture of the Viking Age in Ireland comprising predominantly international conflict but with strong degrees of intranational hostilities too. The principal aims of what follows, then, are (i) to present visualizations for the social and conflictual character networks, (ii) to use the notion of categorical assortativity tailored to estimate where a network of interactions is positioned on the spectrum from the international to the intranational and (iii) to apply that tool to the networks recorded in *Cogadh Gaedhel re Gallaibh*.

## 2. Background

Because *Cogadh Gaedhel re Gallaibh* is a relatively esoteric text (compared with the Greek and Roman classics, for example), in this section, we present a review of existing literature on the topic which it addresses. We also discuss the authenticity and deficiencies of *Cogadh Gaedhel re Gallaibh* as it is used on both sides of the debate. This review therefore serves to contextualize the text and to motivate a new type of scientific study of it.

### 2.1. Context: the war of the Gaedhil with the Gaill

*Cogadh Gaedhel re Gallaibh* comes down to us in three manuscripts. The oldest is in the twelfth-century *Book of Leinster* which contains part of the tale. The second (also incomplete) is the *Dublin Manuscript*, dated to the fourteenth century. The third and only complete text is the *Brussels Manuscript.* This was transcribed from an earlier (now lost) manuscript by the famous Franciscan friar Mícheál Ó Cléirigh who in the seventeenth century was sent from Louvain in Belgium to Ireland to collect and preserve Ireland’s ancient heritage. The Brussels and Dublin manuscripts are close but not identical. Máire Ní Mhaonaigh gives a detailed textual history of *Cogadh Gaedhel re Gallaibh* in [29,30]. As a proxy for the originals, we use the nineteenth-century translation into English by Todd [4]. Todd’s edition, which was 150 years old in 2017, is accompanied by an extensive introduction and by detailed explanatory footnotes. It serves as a source for some scholars wishing to access the narrative today [38]. Todd considered *Cogadh Gaedhel re Gallaibh* as divisible into two parts. The first recounts the arrival and deeds of the Vikings in Ireland in a rough chronological fashion. The second part concerns Brian Boru and his Munster dynasty whose powerbase was on the banks of the river Shannon. The lives and politics of his family are outlined along with numerous encounters with the Vikings, all leading to the events at Clontarf.

Brian Boru was king of the Dál Cais in the northern part of the province of Munster (a map of Ireland during the Viking Age is provided in appendix A). After various battles at provincial level, Brian and the Dál Cais consolidated rule of Munster, defeating their Irish and Norse challengers. Brian then turned his attention to the easterly province of Leinster and the westerly province of Connacht. This brought him into contest with Máel Sechnaill mac Domnaill, king of Meath and most powerful king in Ireland, but in 997, Brian and Máel Sechnaill agreed a truce, whereby the former would rule over the (approximate) southern half of Ireland, while the latter kept the (approximate) northern half. By these means, Brian came to control Munster, the area immediately north of Dál Cais territory in southern Connacht, and Leinster as well as the Hiberno-Norse cities within, while Máel Sechnaill held the province of Meath, part of Connacht with at least a notional claim of authority over the northern part of Ireland.

In 998, Brian and Máel Sechnaill worked together against the Dublin Norse. The Vikings had established a settlement in Dublin in 838 and during the following century they developed a kingdom comprising large areas surrounding the town and controlling parts of the Irish Sea. Viking Dublin was politically linked at various times to the Isle of Man and the Hebrides, as well as to Viking settlements in Britain and Scandinavia. Dublin was joined by Leinster under a new king, Máel Morda mac Murchada, in opposing Brian and Máel Sechnaill. Leinster traditionally rejected the rule of both Munster and Meath and the Hiberno-Norse city of Dublin was ruled by Máel Morda’s nephew, Sigtrygg Silkbeard. The two sides met at Glenmama in late December 999. The Irish annals agree that the combined forces of Munster and Meath decisively defeated those of Leinster and Dublin.

The river Shannon presented a barrier to Meath receiving support from his ally Cathal mac Conchobar mac Taidg, king of Connacht, when Máel Sechnaill came under attack by Brian in the year 1000. By 1002, Máel Sechnaill had submitted to Brian at Athlone [6]. The next target for Brian was the northern kingdoms. It took 10 years, a combination of forces and coordinated use of sea and land attacks, and support from the Church in Armagh for the Northern Uí Néill and regional kings of modern-day Ulster to submit to Brian. By 1011, Brian had achieved his aim of bringing all the regional rulers of Ireland under his control.

In 1012, Máel Mórda mac Murchada of Leinster rose in rebellion. Allied with Flaithbertach Ua Néill, regional king of Ailech in the north-west, he again attacked Meath. Máel Sechnaill sought Brian’s help and the following year Brian and his son led a combined force from Munster and Connacht into Leinster, reaching Dublin in September. Out of supplies near the end of the year, they abandoned their siege of the walled city, with an intention to return.

Thus was the background to the famous Battle of Clontarf. In 1014, Máel Morda’s cousin, Sigtrygg, journeyed to Orkney and the Isle of Man seeking Viking support. These Norsemen came under Sigurd Hlodvirsson (Earl of Orkney, known as Sigurd the Stout) and Brodir, reputedly of the Isle of Man. Brian’s forces came from Munster and southern Connacht possibly supported, at least initially, by Máel Sechnaill’s Meathmen (the precise role of Meath in the battle itself is a matter of some contention [5,7,36]). The Battle of Clontarf is believed to have taken place on Good Friday, 23 April 1014 [4] (see, however, [13,57]). According to the *Cogadh*, after a day’s fighting, the battle ended with the routing of the Viking and Leinster armies. The account tells us that their retreat was cut off by the high tide. Many of the nobles died. Brodir killed Brian, having found the old man in his tent. *Njáls Saga* informs us that Brodir in turn was killed by Úlf Hreða (possibly Cuduiligh in the *Cogadh* [58], meaning Wolf the Quarrelsome), a relative of Brian Boru. Sigurd the Stout of Orkney was also killed, as was the Leinster king Máel Morda mac Murchada. Sigtrygg Silkbeard survived and remained king of Dublin, and the king of Meath, Máel Sechnaill mac Domnaill, resumed his claim to high kingship of Ireland,^6^ supported by Flaithbertach Ua Néill.

### 2.2. Authenticity and deficiencies of *Cogadh Gaedhel re Gallaibh*

It is nowadays widely accepted that one of the main aims of *Cogadh Gaedhel re Gallaibh* was to document the achievements of the Dál Cais and eulogise Brian Boru ‘… to create an illustrious past for his dynasty and to underline thereby later Uí Brian claims to political power’ [29]. Although it is a valuable resource for studies of the Viking Age in Ireland, it is considered a biased one. The question of its reliability has been the topic of a very long-standing debate [4–7,30,48]. Besides some clear interpolation (described in §3.3), much of its bias appears in the descriptive detail of the narrative. Ours, however, is a statistical analysis and, as such, is rather concerned with the totality of the interactions between characters rather than rhetorical levels of detail. As with any statistical analysis, what it delivers is a summary which captures aggregate characteristics, largely insensitive to individual elements. In this sense, one may hope that it delivers useful statistical information on the Viking Age in Ireland.

Estimates for the date of *Cogadh Gaedhel re Gallaibh* are various. Todd stated its author ‘was a contemporary and strong partizan of King Brian’ [4]. Robin Flower also considered the chronicle ‘almost contemporary’ [25]. Albertus Goedheer gives a date as late as 1160 [23] but John Ryan argues that *Cogadh Gaedhel re Gallaibh* ‘might have been composed about 1130 or earlier’ [24]. In [6], Donnchadh Ó Corráin refers to it as ‘written in the twelfth century’. He also describes the hypothesized text known as *Brian’s saga* as written about 1100 in response to *Cogadh Gaedhel re Gallaibh*, a suggestion that implies a date before 1100 for the creation of the latter [6]. More recent scholarship by Ní Mhaonaigh gives the likely composition date of *Cogadh Gaedhel re Gallaibh* as between the years 1103 and 1113 [29]. (She dates the common source for the Dublin/Brussels recension as the 1120s or 1130s [27,29].) Denis Casey also reviews dating estimates in [41] and argues that there may have been multiple versions of the *Cogadh* (see also [48,46]). Seán Duffy believes it may be ‘based on contemporary annals and, no doubt, local memory’ [7]. He suggests that *Cogadh Gaedhel re Gallaibh* gives ‘a vivid picture of what happened at Clontarf as related perhaps to the writer of the Cogadh by a veteran’ and gives the possibility that it ‘was written by someone who may well have lived through these last years of Brian’s life’. This bringing us back to Todd’s original estimate [4].

The interpretation of *Cogadh Gaedhel re Gallaibh* as propagandistic is linked to the question of the date of its composition because ‘Heroic stature presupposes nurturing by time’ [27]. Thus, its propagandistic nature ‘implied that it could no longer be considered contemporary with any of the events it describes’ [27]. The greater the distance between the events of Clontarf and the setting down of *Cogadh Gaedhel re Gallaibh*, the more room there is for a distorted view to take hold. This is the reason why a good estimate date for the composition of the *Cogadh* is important in the present context. Ryan writes: ‘In the course of the eleventh century, … the view seems to have gained universal acceptance that the Battle of Clontarf was par excellence the great decisive struggle of Irish history. Brian in the retrospect was everywhere acclaimed as a national hero’ [5]. The claim is that time distorted reality; ‘The Norse were a substantial section of the opposing force, and in the mellow haze of popular imagination the battle tended to be transformed into a clear-cut issue, Irish versus Norse, with the former victorious. Even in the Northern countries the battle passed rapidly from history into saga’ [5]. The above estimates for the interval between Clontarf and composition of *Cogadh Gaedhel re Gallaibh* range between contemporary and about 150 years. Our approach cannot deliver an independent estimate for the date of composition and the above estimates should be kept in mind. While the above considerations suggest that the *Cogadh* may distort in favour of an overly international picture of conflict (and, indeed, the contemporary name of the tale itself emphasizes the Viking–Irish conflict), on the other hand it should also be kept in mind that, in places, it identifies Leinster as the principal enemies of Brian [7,13].

In his Introduction to *Cogadh Gaedhel re Gallaibh*, Todd acknowledges the defects of the work and expresses regret that it is ‘so full of the feelings of clanship, and of the consequent partisanship of the time, disfigured also by considerable interpolations, and by a bombastic style in the worst taste …’. In chronicle literature, an interpolation of the type mentioned by Todd is a later addition not written by the original author. We address this issue in §3.3.

Ó Corráin states that the author of *Cogadh Gaedhel re Gallaibh* ‘drew his material from the extant annals but he telescoped events, omitted references to other Viking leaders and concocted a super-Viking, Turgesius, whose wholesale raiding and, particularly, whose attack on Armagh was intended to demonstrate the inefficiency of the Uí Néill as defenders of the church and of the country in contrast of the achievements of the great Brian’ [6]. (Turgesius is elsewhere referred to as ‘exaggerated’ rather than ‘concocted’ [43].) Clare Downham states that throughout the *Cogadh*, ‘records of alliances between Vikings and Irish rulers are neglected; a number of victories won by rulers other than Uí Bhriain are omitted’. Moreover, ‘paired names of Vikings rhyme or alliterate and do not transfer easily into Old Norse equivalents …. These names look as if they have been invented by the author … or drawn from a poetic source’ [48]. Downham further suggests that since ‘historical accuracy, according to the modern definitions, was not the priority’ in *Cogadh Gaedhel re Gallaibh*, ‘the material which is unique to that narrative deserves to be treated with some caution’ [48].

Duffy, on the other hand argues that, whatever about the detail of *Cogadh Gaedhel re Gallaibh* ‘and its slightly cavalier approach to chronology’, the gist of the account ‘seems sound’ [7]. Duffy also discusses difficulties in using the annals to check the historicity of *Cogadh Gaedhel re Gallaibh*. By his reckoning, although some of the names of individuals drafted in from beyond Ireland are indeed suspicious, ‘up to half of them appear to be real and their presence at Clontarf is historically credible, if not corroborated by some other source’ [7]. In [30], Ní Mhaonaigh shows that genuine annals underlie *Cogadh Gaedhel re Gallaibh* and that the compiler of *Cogadh Gaedhel re Gallaibh* ‘remained fairly true to his exemplar’. ‘Provided, therefore, that we keep the redactor’s political purpose firmly in view, we may tentatively add the annalistic material preserved in *Cogadh Gaedhel re Gallaibh* to our list of sources for information on the history of Ireland in the Viking Age’ [30].

Todd himself also reports what he considers to be ‘curious incidental evidence’ for reliability of at least some of the *Cogadh* account in that it ‘was compiled from contemporary materials’ [4]. ‘It is stated in the account given of the Battle of Clontarf, that the full tide in Dublin Bay on the day of the battle (23rd April, 1014), coincided with sunrise’ [4]. In a piece of ‘mathematical detective-work’ [7] that precedes our own by 150 years, Todd’s colleague established that the full tide that morning occurred at 05.30 and indeed coincided with sunrise. For Todd, this ‘proves that our author, if not himself an eye-witness, must have derived his information from those who were’ [4]. We have already seen the importance of the time of the evening tide; calculated to have been at 17.55, consistent with the account in *Cogadh Gaedhel re Gallaibh*; it prevented the escape of the Viking forces and considerably aided Brian’s victory. (See [57] for a recent discussion on this topic.)

This is certainly among the most striking evidence in support of the account of *Cogadh Gaedhel re Gallaibh*. Duffy provides multiple other instances where the *Cogadh* may be reliable [7]. Certainly bombastic statements that are not backed up by the annals have to be treated warily. But notwithstanding this, he considers the narrative as having ‘some credibility’, although ‘unreliable in its precise detail’ [7]. (For criticism of Duffy’s counter-revisionist views, see e.g. [13].)

To summarize, there is a vast amount of humanities scholarship concerning *Cogadh Gaedhel re Gallaibh*. Although some dispute its reliability, others consider its version of events mainly credible and largely consistent with other sources and evidence. As stated by Duffy, ‘even though it is exaggerated and biased’, *Cogadh Gaedhel re Gallaibh* can be useful ‘if we use it judiciously’ and ‘make allowance for its propagandist tendency’. The composer surely did not think in terms of network science but, in recording a cast of hundreds connected with well over a thousand links between them, he nevertheless imprinted networks in the narrative. (We explain how we harvest these data in §3.1.) Thus, we may expect that the bulks of the networks contained in *Cogadh Gaedhel re Gallaibh* might not be too far away from the reality of the networks of the Viking Age in Ireland. Many of the objections listed above are largely irrelevant to our approach as static networks are immune to ‘bombastic’ descriptions, ‘telescoping’ of events and ‘cavalier’ attitudes to chronology. We will see that the aggregate approach is even resistant to isolated cases of interpolation. It is with this perspective that we interrogate the narrative with a networks-science methodology. To recap, our primary aim is to determine whether the character networks in *Cogadh Gaedhel re Gallaibh* are implicative of an ‘international contest’ or ‘local quarrel’ [12].

### 2.3. International contest or local quarrel?

O’Connor [2] in the eighteenth century, with Ryan [5] and Ó Corráin [6], in the twentieth, are considered early debunkers of the traditional myth of Clontarf [29,7]. O’Connor describes the conflict as a ‘civil war’ in which ‘the whole province of Leinster revolted, and called the Normans from all quarters to its assistance’ [2]. Ryan’s main claim is that ‘In the series of events that led to Clontarf it was not … the Norse but the Leinstermen, who played the predominant part’ [5]. His thesis is that the conflict is not a ‘clear-cut’ one between Irish and Viking. Firstly, Brian’s army was not a national one, but one of Munstermen supported by two small Connacht states. Secondly, the opposition ‘was not an army of Norse, but an army composed of Leinster and Norse troops, in which the former were certainly the predominant element and may have constituted two-thirds of the whole’ [5]. The battle, then, was not a contest for the sovereignty of Ireland—it was not a clear-cut issue of Irish versus Norse. Instead, the issue at hand was ‘the determination of the Leinstermen to maintain their independence against the High-King’ [5].

It was in the course of the eleventh century, Ryan argues, that the picture of a decisive struggle of Irish history gained ‘universal acceptance’ in the popular imagination. This came about because of the parts played by forces from the Isle of Man and the Orkney Islands together with the partisan nature of *Cogadh Gaedhel re Gallaibh*. It was only in this retrospect that Brian was acclaimed as a national hero. Ó Corráin’s view is similar [6]: ‘The battle of Clontarf was not a struggle between the Irish and the Norse for the sovereignty of Ireland …. [It] was part of the internal struggle for sovereignty and was essentially the revolt of the Leinstermen against the dominance of Brian, a revolt in which their Norse allies played an important but secondary role’.

Duffy points out that this revisionist interpretation is not supported by the other ancient annals. For example, the Annals of Inisfallen gives a short but reliable account ‘reflective of contemporary reaction to what occurred’ [7]. It is stated that ‘the Foreigners of Dublin gave battle to Brian’ and Leinstermen are also slain. According to Duffy, ‘Whereas some modern historians see the Leinstermen as Brian’s primary enemy at Clontarf, the annalist was in no doubt that the enemy was the Norse of Dublin. In fact he has the same black-and-white picture of the opposing sides that we tend to think of as later legend …’. ‘The entry in the Annals of Ulster also echoes the Annals of Inisfallen in emphasizing the primacy of the Norse as Brian’s adversaries’. Duffy states that the Annals of Ulster suggest ‘it was fundamentally a contest between the Irish and Norse (although the latter too had Irish allies)’.

Duffy provides multiple items of evidence in support of his view that ‘Brian’s principle opponents were the Hiberno-Norse allied to Leinster’ and that the Battle of Clontarf ‘was notable in particular for the great numbers of overseas Norse forces present, and for the huge losses they incurred by fighting and drowning’. ‘Implicitly, for the Cogadh’s author, two centuries of Irish opposition to Viking invasion, spearheaded by Brian’s dynasty, reached a climax at Clontarf. That picture was imprinted too, with remarkable correspondences, on the minds of … thirteenth-century Icelandic writers. Those who did battle with Brian came from the Norse world seeking a kingdom for themselves in Ireland’.

Thus, the debate about Clontarf has spanned the centuries and frames our present investigation. Here, we broaden the question to how conflictual and social relationships are presented in *Cogadh Gaedhel re Gallaibh*.

## 3. Methods: the *Cogadh* narrative network

In this section, we explain the methods by which the data were harvested and our focus on network topology. We also present a visualization of the *Cogadh* narrative network and discuss how interpolation has negligible effect on our network statistics. To keep the main text manageable, we defer details concerning various assortativity measures to appendix B and the roles played by the most important characters to appendix C along with an analysis of network robustness.

### 3.1. Constructing the *Cogadh* network

As with previous studies [49–51,60,61], we consider *Cogadh Gaedhel re Gallaibh* as playing out on a complex network comprising *N* nodes and *M* edges. The edges link the nodes through relationships or interactions. We distinguish between three categories—Irish, Viking and other—identifying to which group each node belongs from the text itself. We obviously cannot directly access the reality behind the text to determine any gradation between the groups. For example, we cannot know how Sigtrygg Silkbeard, who had a Viking father and an Irish mother, might have self-identified in reality; we can only take our lead from the *Cogadh* itself and since the Hiberno-Norse of Dublin are presented there as Vikings, they are placed in that category. Nodes classified as ‘other’ are those that are not readily assigned to either camp.

Our approach to constructing the networks follows the methodology of [49–51] in that nodes and links are identified by carefully and manually reading the texts with multiple passes through all of the material by multiple readers. In our experience, such an approach is required to minimize errors and omissions as well as to reduce levels of subjectivity. *Cogadh Gaedhel re Gallaibh* is a very dense text and meticulous care is required to interpret extremely subtle tracts containing large amounts of explicit and implicit information. It is currently beyond technological capabilities to extract such information automatically owing to the inherent complexity of such texts (see, e.g. [62]). Establishing the technology for such an approach is another active area of research.

Figure 1 contains a network visualization of the full set of interactions recorded in the *Cogadh*. Green nodes represent Irish characters and green edges represent interactions between them. The counterpart set of Viking nodes and their interlinks are in blue. Brown edges represent interactions between Irish and Viking nodes. Any remaining nodes and edges are in grey.

We distinguish between two types of edge: positive and negative. Positive edges are established when any two characters are related, communicate directly with each another, or speak about one another, or are present together when it is clear that they know each other. So positive edges ordinarily represent familial or social relationships. Negative links, on the other hand, are formed when two characters meet in physical conflict or when animosity is explicitly declared by one character against another and it is clear they know each other (such as a declaration of war). So negative edges typically represent actual or intended physical hostility. It is possible that two characters are linked by both positive and negative edges as relationships between characters may change over time.

Ours is a static analysis, capturing the temporal totality of the *Cogadh* narrative. ‘Making the past just as visible as the present’, as Moretti puts it [63], is a benefit of this networks approach and one which has been used elsewhere [49,50]. Nonetheless, it should be noted that the study of dynamical properties of networks constitutes an active, broad and developing area of research and such an approach would be of interest in the future [61]. We focus primarily on the *topology* of the networks underlying *Cogadh Gaedhel re Gallaibh*, considering undirected, unweighted networks. This means that (i) the features which connect the various nodes are not oriented and (ii) the statistics we report upon do not take into account varying levels of intensity of interactions between nodes. To account for (i), one would have to introduce a level of detail which is finer that just positivity or negativity. However, what one gains in refining details, one loses in statistical power. To account for (ii), one may place higher weight on more intense interactions, but, besides using the number of interactions between characters in the narrative, there is no established standard mode of weighting edges in character networks. Moreover, we are primarily interested in the presence or absence of conflict, not on the details of varying intensity of such hostility. Therefore, we defer consideration of directed, weighted and temporal networks for future studies and restrict the current study to network topology and related matters.

### 3.2. Network methodology: basic statistics

We identified *N*=315 individual interacting characters in Todd’s translation of *Cogadh Gaedhel re Gallaibh*.^7^ These nodes are interconnected by *M*=1190 edges and we refer to the corresponding assemblage as the *entire* network. We can also consider the positive and negative sub-networks, formed only of positive or negative edges, respectively. Examination of these allows us to gain more insight into the social and conflictual statistics contained in the narrative. Indeed, it is long known from sociology that societies exhibit *homophily*, the tendency of individuals to associate with others who are similar to themselves [64–66]. In the field of social network analysis, this is known as assortativity. In previous studies of epic literature [49–51,60], we studied *degree assortativity*, the tendency (or otherwise) of nodes to attach to other nodes with similar numbers of links. We found some positive sub-networks exhibit degree assortativity, or are uncorrelated, while the opposite feature—degree disassortativity—is characteristic of negative sub-networks. This means that positive social networks give a ‘cleaner’ picture (relative to full networks) of the non-conflictual societies underlying such narratives, making it valuable to study them in isolation [67]. A new feature of the current study is our additional focus on the negative sub-network to statistically measure levels of hostility.

We use the term *unsigned* to refer to networks containing both positive and negative edges. Networks comprising only positive (or only negative) edges are then themselves termed *positive* (or *negative*, respectively). We use the term *full-cast* to refer to networks containing the full cast of characters, Irish, Viking and others. Networks containing only Irish (or only Viking) characters are themselves referred to as *Irish* (or *Viking*, respectively). This terminology is summarized in table 1. Statistics for the entire network and various sub-networks are collected in table 2.

**Table 1.** Full-cast networks comprise Irish, Viking and other nodes together with interactions between them. Unsigned networks comprise positive and negative edges as well as the nodes they connect. Thus, for example, the positive, full-cast network comprises all nodes but only positive links. The unsigned, Irish network comprises only Irish nodes but both positive and negative links between them. The *entire* network comprises all interacting nodes and all links.

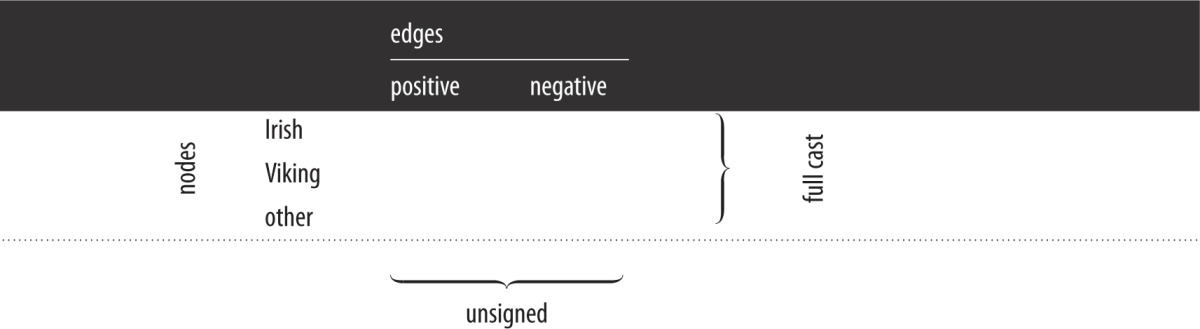

**Table 2.** Statistics for the entire network and its various sub-networks. The first and second columns indicate whether the sub-network is unsigned, positive or negative with full cast of characters (Irish, Viking and other) or only the Irish or Vikings are taken into account. Here, *N* represents the number of nodes; *M* is the number of edges; 〈*k*〉 is the mean degree and *k*_max_ its maximum. The proportion of triads that contain an odd number of positive links is represented by Δ and the degree assortativity is denoted by *r*.

		*N*	*M*	〈*k*〉	*k*_max_	Δ	*r*
unsigned	full cast	315	1190	7.6	105	0.93	−0.09(2)
	Irish	193	530	5.5	63	0.93	−0.08(3)
	Vikings	91	313	6.9	26	1.00	0.31(7)
		*N*^+^	*M*^+^	〈*k*〉^+^	*k*^+^_max_		*r*^+^
positive	full cast	287	957	6.7	53		0.00(4)
	Irish	186	475	5.1	47		−0.02(4)
	Vikings	88	301	6.8	26		0.34(7)
		*N*^−^	*M*^−^	〈*k*〉^−^	*k*^−^_max_		*r*^−^
negative	full cast	180	264	2.9	63		−0.25(3)
	Irish	62	72	2.3	25		−0.26(6)
	Vikings	18	16	1.8	4		−0.08(18)

The average number of edges per node for the entire network is 〈*k*〉=2*M*/*N*≈7.6. The actual number of edges associated with the *i*th node is denoted by *k*_*i*_. This is a number which varies between 1 for the least connected characters (nodes with *k*_*i*_=0 have no links and are not attached to the network at all) and *k*_max_ for the most connected (in a sense, the most important) character. For the entire network, the most connected character is Brian himself who, with *k*_max_=105 edges, is linked to 33% of the other characters in the narrative. Besides Brian’s degree, we are also interested in the connectedness of other characters and we rank the first few characters according to their individual degrees, and according to other measures of importance, in appendix C.

*Cogadh Gaedhel re Gallaibh* has *N*^+^=287 interacting characters in its positive sub-network, interconnected by *M*^+^=957 edges, corresponding to a mean degree of 〈*k*〉^+^≈6.7.^8^ Here and henceforth, we use the superscripts ‘+’ and ‘−’ to identify statistics associated with the positive and negative networks, respectively. (We omit such a superscript from statistics for the unsigned networks. These are distinguished from generic symbols by context.) The counterpart figures for the negative network are *N*^−^=180, *M*^−^=264 and 〈*k*〉^−^≈2.9, respectively. (The total number of positive and negative links *M*^+^+*M*^−^=957+264=1221 exceeds the number *M*=1190 which we previously identified for the entire network because some relationships involve both positive and negative aspects.) As for the entire network, Brian has the highest degrees in both positive and negative subgraphs, with the former measured at *k*^+^_max_=53 and the latter at *k*^−^_max_=63.

The adage that ‘the enemy of an enemy is a friend’ is related to the notion of *structural balance* in network science [68–70]. The maxim suggests that *triads* (sets of three mutually connected nodes) with one positive and two negative edges are commonplace. More generally, triads with odd numbers of positive edges are considered structurally balanced. One way to quantify the extent to which it holds in a character network is through the statistic Δ, defined as the percentage of triads that contain an odd number of positive links. A large value of Δ means that hostility between two characters is suppressed if they have a common foe. Clearly Δ is only meaningful for the unsigned network; on the positive sub-network it is 1 by definition, while in the negative sub-network it is necessarily zero. We find that the entire network underlying *Cogadh Gaedhel re Gallaibh* (which has 3041 triads) is indeed structurally balanced with Δ≈93%.

As mentioned above, assortativity (disassortativity) is the tendency for the nodes of a network to attach to other nodes that are similar (different) in some way. Network theorists frequently measure *degree assortativity*—the extent to which nodes of similar degree tend to link up. As with other character networks, we find that the negative full-cast network is disassortative by degree *r*=−0.25(3).^9^ This means that high-degree characters are hubs and their negative links preferentially attach to low-degree ones. This appears to be a generic feature of heroic tales in particular, where the hero or heroes encounter multitudes of lesser characters and defeat them in battle. The positive full-cast network, on the other hand, is uncorrelated within errors (*r*=−0.00(4), meaning it is neither assortative nor disassortative). These features are typical of social networks and of character networks with positive interactions [67,49].

Besides the networks comprising the full cast of characters, we can also consider the networks containing only Irish or only Viking nodes and these are also listed in table 2.^10^ We observe the following average properties of the various networks. In the Irish and Viking networks (as in the full-cast cases), the mean degrees are maximal for the unsigned networks and minimal for the negative sub-networks. The unsigned Viking network is more structurally balanced than its Irish counterpart. Structural balance for the Irish network, which has 830 triads, is 93% whereas the 881 Viking triads all contain odd numbers of positive links.

### 3.3. Effect of interpolation on network statistics

In his Introduction to *Cogadh Gaedhel re Gallaibh*, Todd acknowledges the defects of the work and expresses regret that it is ‘so full of the feelings of clanship, and of the consequent partisanship of the time, disfigured also by considerable interpolations, and by a bombastic style in the worst taste …’. In chronicle literature, an interpolation of the type mentioned by Todd is a later addition not written by the original author. As scribes copied ancient material by hand, extraneous material frequently came to be inserted for a variety of reasons [71]. These may have been for bona fide intentions, perhaps as explanations; for subjective purposes; or they may simply have crept in through errors and inaccuracies arising from manual copying or, indeed, as attempts ‘to enhance the appeal of the narrative’ [27]. One way to detect such interpolation is through comparing different manuscripts.

Perhaps the most famous interpolation in the narrative is a passage which occurs in the Dublin version describing the actions of Fergal Ua Ruairc of Bréifne and associate chieftains [5,27]. (For the location of Bréifne, see figure 3.) The Brussels manuscript, by contrast, ‘omits everything connected with Fergal and his presence in the battle’ [4]. As stated by Todd, ‘the whole story bears internal evidence of fabrication, for Fergal O’Ruairc was slain AD 966 …, and our author had already set him down among Brian’s enemies’. Ryan [5], Duffy [7] and others also identify Ua Ruairc as an interpolation and Ní Mhaonaigh gives a detailed account of Bréifne bias in *Cogadh Gaedhel re Gallaibh* [27]. She states ‘one of the main aims of the interpolator was to portray Fergal Ua Ruairc and his followers in as favourable a light as possible, sometimes regardless of the effect this had on his text’. The point is that a pro-Ua Ruairc reviser of the narrative may have deemed it politically expedient to alter the record of relations between the Uí Ruairc and the Dál Cais by demonstrating assistance given by the former to Brian at Clontarf. Ní Mhaonaigh estimates the period when the Uí Ruairc were likely to have gained maximum advantage from such an association to have been the mid- to late 1140s, over a hundred years after Clontarf [27].

We are interested in what insight the networks methodology can give on such matters. We have already seen that 93% of the 3041 triads in the unsigned network are structurally balanced as are 93% of the 830 triads in the Irish network. The triad formed by Ua Ruairc’s enmity to Máel Sechnaill, the latter’s alliance with Brian, and the interpolated support of Ua Ruairc for Brian is one of two positive edges and one negative one, which is structurally *imbalanced*. Since the vast majority of triads in *Cogadh Gaedhel re Gallaibh* are balanced, this makes the Ua Ruairc episode stand out as relatively unusual. We removed Ua Ruairc and his three associates (Gilla-na-Naomh, Mac an Trin and Domhnall mac Raghallach [4]) from the networks to test the effects on the statistics. Besides reducing the number of edges (e.g. *M* reduces from 1190 to 1146 in the entire network), the effects of this removal are minimal. For example, the degree assortativies are unchanged within error estimates for the unsigned, positive and negative networks.

The possibility of interpolation applies not only to Ua Ruairc and allies. Ryan claims that ‘Many of the names mentioned are names only, for nothing is known of the persons who bear them. Some of the levies in important positions were certainly absent. In a word, no effort is made to distinguish between the genuine and the spurious, to criticise suspect sources, and to reconcile contradictions’ [5]. Given the minor effect of the most famous and easily identified, Ua Ruairc, interpolation, we do not attempt to remove other interpolations from our analysis. Besides, any attempt to do so would be incomplete because we cannot be certain that all interpolations have been identified. Indeed, as we have repeatedly emphasized, ours is a network study of *Cogadh Gaedhel re Gallaibh* as represented by Todd in [4] and therefore we present it in its entirety. However, we attempt to simulate the effects of interpolation by randomly removing up to 15% of nodes or edges. The process is repeated 1000 times and the averages deliver no appreciable difference to the statistics given in table 2, indicative of their robustness (see appendix C for a network-robustness analysis). For example, removal of 15% of the vertices alters the assortativity from *r*=−0.09 to *r*=−0.08 (imperceptible change within errors). Removal of 15% of the edges leaves *r* unchanged within this level of precision. A more systematic and targeted quantitative study of the effects of interpolation would be interesting for future study.

## 4. Results: the relationships between Irish and Vikings as recorded in the *Cogadh* networks

The traditional ‘memory’ of the events leading up to the Battle of Clontarf is of an international conflict between two distinct sides: Irish versus Viking [5]. This is dismissed by revisionist historians who argue that the conflict is primarily Irish-on-Irish [2,5,6]. The traditional viewpoint of a clear-cut contest might be expected to lead to a network in which the bulk of negative (conflictual) edges correspond to Irish–Viking interactions representing the primacy of hostility being between the two groups. We might expect a network supporting the revisionist stance to be somewhat different: the negative edges would mainly link Irish nodal pairs. We also have to monitor Viking-on-Viking conflict as there were different Viking factions in Ireland during this period [4,44].

In table 3, we record the proportions of Irish, Viking and other nodes in the unsigned networks and in its positive and negative sub-networks.^11^ At 61%–65 %, the proportions of Irish nodes in each of the three graphs are approximately constant. The proportion of Viking nodes is also relatively stable between 31% and 34%. In the same table, we list the proportions of interactions which link Irish to Irish nodes; Viking to Viking; and Irish–Viking pairs. Fifty percent of edges in the positive network link pairs of Irish nodes; 31% connect pairs of Viking nodes; and 12% of positive interactions connect mixed Irish–Viking pairs. Twenty-seven percent of links in the negative network connect Irish to Irish nodes; 6% connect pairs of Viking nodes; and over 62% of negative interactions connect mixed Irish–Viking pairs. In other words, the positive (social) network is dominated by interactions between characters of the same narrative identities (intranational interactions) and the negative (conflictual) network is dominated by Irish–Viking (international) interactions. This suggests that the largest proportion of *Cogadh* conflict is international, but there are significant levels of intranational hostilities too (especially Irish versus Irish). Actually, from table 3, we see that the number of international edges in the negative network is over twice the number of Irish–Irish negative edges, which, in turn is over four times the number of Viking–Viking negative edges.

**Table 3.** Identity profiles of the cast and their interactions in *Cogadh Gaedhel re Gallaibh*. The second, third and fourth rows give the numbers (and percentages) of nodes which are identified as Irish, Viking and other (not identified as Irish or Viking) in the entire, unsigned network as well as in the positive and negative sub-networks. The fifth row gives the total number of nodes in each network (these values are *N*, *N*^+^ and *N*^−^ for the full-cast networks, respectively). The sixth and seventh rows give the numbers (proportions) of edges which connect pairs of like nodes. The eighth row gives the numbers (proportions) of edges which connect Irish and Viking nodes. The last row gives the total numbers of edges in each case as (*M*, *M*^+^ and *M*^−^ for the full-cast networks). The remaining edges involve other (not assigned as Irish or Viking) nodes.

	entire network	positive network	negative network
Irish nodes	202 (64%)	187 (65%)	110 (61%)
Viking nodes	97 (31%)	88 (31%)	61 (34%)
other nodes	16 (5%)	12 (4%)	9 (5%)
**total # nodes**	315 (100%)	287 (100%)	180 (100%)
Irish–Irish edges	530 (45%)	475 (50%)	72 (27%)
Viking–Viking edges	313 (26%)	301 (31%)	16 (6%)
Irish–Viking edges	272 (23%)	119 (12%)	163 (62%)
**total # edges**	1190 (100%)	957 (100%)	264 (100%)

However, to properly evaluate the levels of mixing, negative or positive, between Irish and Viking, one has also to account for the fact that they do not have the same numbers of nodes in the networks (there are twice as many Irish nodes as Viking). To do this, we introduce the *categorical assortativity* of the various networks, represented generically by *ρ*. Its precise definition is given in appendix B. It is a measure which ranges between *ρ*_min_ and 1 where *ρ*_min_ is a non-trivial, negative value, which itself lies between −1 and 0 if there are more than two categories under consideration [65,66]. Thus, although the maximum value of *ρ* is one, its minimum value can be network dependent. The reason for this is that, when there are more than two categories, disassortativity connects dissimilar nodes, just as randomness does. Assortativity, however, connects like nodes and is therefore quite different to randomness. We have to be mindful of this asymmetry when interpreting the categorical assortativity for the negative networks with three categories of node (Irish, Viking and unassigned). The only instance in which *ρ*_min_=−1 is when there are two categories.

The value *ρ*=1 indicates 100% categorical assortativity. If this were the case for our positive network, for example, it would mean that the only positive interactions are *within* rather than between categories (friendly interactions would be intranational). The value *ρ*=*ρ*_min_<0 implies that the network is fully categorically disassortative. If this were the case for our positive network it would mean that the only positive interactions are *between* rather than within categories (positive interactions would be international). A value *ρ*=0 would indicate that the categorical assortativity is the same as would be expected for random mixing between nodes, oblivious of their Irish or Viking character. We find that *ρ*^+^=0.65(3) for the full-cast positive network. If we restrict our attention to Irish and Viking nodes only by removing other nodes, this rises to *ρ*^+^=0.72(3). These statistics are recorded in table 4 and support the picture that most (but not all) positive interactions are intranational.

**Table 4.** Categorical assortativities. The first column identifies whether all nodes (Irish, Viking and other) are included in the determination of *ρ* or if the unassigned (other) nodes are excluded. In the former case, *ρ*_min_ is determined by equation (5). In the latter case, it is −1. The second column identifies whether all remaining links are included or whether Viking-on-Viking edges are omitted.

nodes	edges	positive network (*ρ*^+^)	negative network (*ρ*^−^)
all nodes included	include all edges	0.65(3)	−0.32(6)
	omit Viking-on-Viking edges	—	−0.45(5)
	*ρ*_min_	−0.62(3)	−0.88(4)
other nodes omitted	include all remaining edges	0.72(3)	−0.37(6)
	omit Viking-on-Viking edges only	—	−0.53(4)
	*ρ*_min_	−1	−1

We now focus our attention on the negative networks as these connect with the debate in the humanities discussed in §2. A ‘clear-cut’ version of the ‘international-conflict’ picture would be characterized by the value *ρ*^−^≈*ρ*^−^_min_ (where *ρ*^−^_min_ is the minimum possible value of *ρ*^−^, and is −1 when unassigned nodes are excluded). Such a value would reflect a purely Irish-versus-Viking conflict. At the opposite end of the spectrum would be a world in which all conflict is intranational. In this case one would expect *ρ*^−^≈1. The revisionist picture of a primarily (but not exclusively) intranational conflict may be expected to correspond to a positive value of *ρ*^−^. Between the two extremes, we might imagine a more even distribution of negative edges, whereby conflict between nodes is ‘blind’ to their identities. A completely colour-blind narrative would deliver *ρ*^−^≈0 for the negative network.

**Figure 2.**
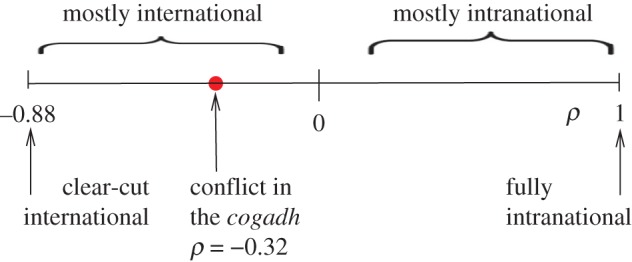
Graphical representation of the main conclusion of this paper. The spectrum of values of categorical assortativity for networks of the conflictual- *Cogadh* type ranges from *ρ*=−0.88 to *ρ*=1. Negative values of *ρ* correspond to various degrees of the traditional picture of international hostilities with *ρ*=−0.88 representing a clear-cut Irish-versus-Viking conflict. Positive values correlate with the revisionist picture of mostly intranational conflict. The analysis presented in this paper shows that the *Cogadh* hostile network delivers a value −0.32 which, although not clear-cut, lies on the traditional side of the spectrum.

**Figure 3.**
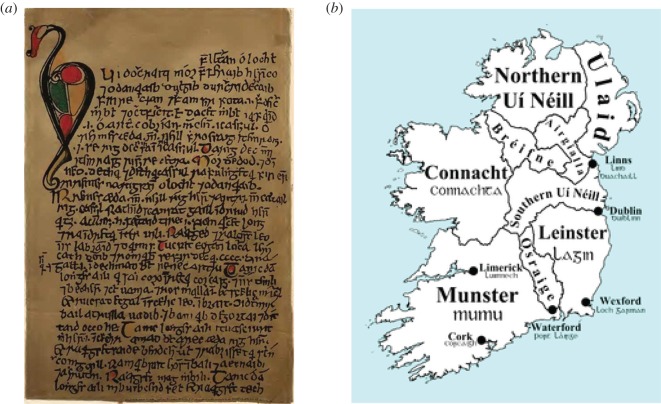
(*a*) Image of the nineteenth-century facsimile of the opening page of *Cogadh Gaedhel re Gallaibh* which was reproduced in Todd’s edition [4]. (*b*) The main kingdoms of Ireland *ca* AD 900 with principal (Viking) towns.

We find that *ρ*^−^=−0.32(6) if all three kinds of node (Irish, Viking and other) are included in the negative network. This statistic is to be compared to the theoretical minimum *ρ*^−^_min_=−0.88(4). If unassigned nodes are omitted, one finds *ρ*^−^=−0.37(6) (with *ρ*^−^_min_=−1). Thus our measured values for categorical assortativity on the negative (conflictual) networks are themselves negative. This means that the picture of a primarily intranational conflict is not supported by data contained in *Cogadh Gaedhel re Gallaibh*. However, the conflict is not clear-cut international either; it is a narrative in which the highest proportion of conflict is presented as being between Irish and Viking but with significant amounts of green-on-green and blue-on-blue conflict too. On the spectrum from international to intranational conflict, representing various degrees of the traditional to the revisionist views, the negative *Cogadh* networks are firmly on the traditional side but at a moderate and not a limiting value. This spectrum is represented graphically in figure 2. This is the main conclusion of our paper and is our contribution to the 250-year-old debate mentioned in the Introduction.

The assortativity analysis thus far probes the extent to which conflict or harmony reigns within or between the two groups. However, one may argue that the revisionist concern is with the Irish side. The claim is that the conflict is primarily within the Irish community—not that it is both within the Irish cast and within the Viking set. Clearly, there was a great degree of such conflict too; e.g. Ryan states ‘The Norse were traditionally unscrupulous in preying upon one another’ [5]. (See also [47].) Therefore, one may argue that Viking-on-Viking conflicts could contaminate our measurements. Our aim is to determine whether the Irish are mostly in conflict with other Irish or with Vikings; in this sense, the fact that the Vikings were also fighting among themselves is irrelevant.

To investigate further, we remove all Viking-on-Viking links from the negative sub-network. Recalculating the categorical assortativity delivers *ρ*^−^=−0.45(5) (*ρ*^−^=−0.53(4) if the unassigned nodes are removed) which indeed is larger in magnitude than the previous measure (the assortative Viking-on-Viking edges having been removed). But it is still not a clear-cut Irish-versus-Viking picture; i.e. it is not close to *ρ*^−^_min_=−0.88(4) (or −1 in the case where unassigned nodes are removed). Thus our conclusions are unchanged. These statistics are listed in table 4.

In appendix B, to overcome the awkwardness of network-dependent $\rho_{\min}$-values, we introduce a renormalized categorical assortativity measure that ranges from −1 in the case of fully disassortative networks through zero for uncorrelated networks to 1 for fully assortative networks. We also present in table 5 an alternative to table 4, using these renormalized values.

**Table 5.** The set of renormalized categorical assortativity values $\hat{\rho }$ from equation (6) presented here is an alternative to table 4. Fully disassortative, uncorrelated and assortative networks have $\hat{\rho }=-1$, $\hat{\rho }=0$ and $\hat{\rho }=1$, respectively.

nodes	edges	positive network (${\hat{\rho }}^{+}$)	negative network (${\hat{\rho }}^{-}$)
all nodes included	include all edges	0.65(3)	−0.32(6)
	omit Viking-on-Viking edges	—	−0.43(5)
	*ρ*_min_	−1	−1
other nodes omitted	include all remaining edges	0.72(3)	−0.33(6)
	omit Viking-on-Viking edges only	—	−0.44(5)
	*ρ*_min_	−1	−1

In summary, we conclude that the character networks embedded in the *Cogadh Gaedhel re Gallaibh* do not support clear-cut traditionalist or revisionist depictions of the Viking Age in Ireland. Instead they support a moderate traditionalist picture of conflict which is mostly between Irish and Viking characters, but with significant amounts of hostilities between both sides as well.

## 5. Discussion

The popular tradition associated with the Viking Age in Ireland and the events of Clontarf in 1014 is that Brian’s principal opponents were Vikings. Following Charles O’Connor in 1766, in 1938 John Ryan [5] published what has been described as an ‘assault’ [12] on that traditional interpretation. Instead of a ‘clear-cut’ Irish versus Norse conflict, the revisionist claim is that it was a struggle primarily between Irish forces. With the millennial anniversary of the Battle of Clontarf, Seán Duffy attacked ‘the new orthodoxy’ [12] and launched a counter-revisionist defence of the traditional picture [7]. His judicious use of *Cogadh Gaedhel re Gallaibh* and other texts leads him to conclude that ‘The Battle of Clontarf was an international contest’ [12]. This view has itself come in for criticism [13] and the anniversary reinvigorated lively discussions and healthy debate among experts and the wider public. This and the 150th anniversary of Todd’s famous translation [4] form the context in which the above results are presented.

*Cogadh Gaedhel re Gallaibh* is a skilfully written propagandistic text, replete with bias, exaggerating virtues and vices of many of its characters [4,27,30]. It has been used to support arguments from both sides of the debate. Duffy describes it as a ‘long narrative of Irish conflict with the Vikings’ [7]. Downham states ‘Evidently the conflict was much more than an internal squabble between an Irish over-king and some reluctant subjects’ [18]. Etchingham, on the other hand, in reviewing [7], stresses that ‘even Cogadh actually identifies the Leinstermen as principal rebels’ [13]. From the side opposing Brian at Clontarf, the *Cogadh* gives the majority of the slain (3100 out of 5600) as Irish [4,5], tallies which could be viewed as supporting the picture of a mostly domestic conflict. At least these tallies show that *Cogadh Gaedhel re Gallaibh* does not pretend that Viking slain exceed the numbers of Leinstermen in order to ‘internationalize’ the story. This may suggest that, interpolations notwithstanding [48], even if the *Cogadh* exaggerated qualities, it may not have exaggerated quantities (at least not by much). Indeed, Ryan believes that the account of the actual battle of Clontarf in the *Cogadh* is ‘incomparably the most reliable’.

In the above considerations we have gone beyond a simple tally of the slain and performed a character-network analysis of *Cogadh Gaedhel re Gallaibh*. Since this is wholly independent of the tone of the account (‘bombastic’ and ‘partisan’) and its shortcomings (‘telescoping’ of events and ‘cavalier’ attitude to chronology), we considered this approach a judicious use of the text. To contribute to the debate as to the nature of the Viking Age in Ireland as set down in the *Cogadh*, we applied a measure of categorical assortativity which is capable of taking proportions of Irish and Viking nodes into account. As we have stressed throughout, any statistical analysis is only as good as the data it draws upon and here all of our data comes directly from the *Cogadh* text. Any conclusions about the implications of our study for the reality of the Viking Age in Ireland have to be made in combination with knowledge from humanities literature on the topic. Humanities scholars agree that, to some degree, historical sources lie behind the *Cogadh*. But they differ as to their extent. If, having assessed the evidence, one believes *Cogadh Gaedhel re Gallaibh*, in the main, to be unreliable, invented or concocted then little can be drawn from our study about reality. Even in this case, however, the text (and hopefully this paper) still delivers information on how medieval writers sought to, or were able to, portray the composition of societies.

A less doubtful assessment of the evidence may offer hope that a reasonable proportion of characters and their interactions reflect the reality of the age (and we have seen that our network statistics are robust; even omitting Viking–Viking interactions does not alter the broad conclusions of our study). Indeed, since the *Cogadh* author scarcely anticipated a complexity-scientific analysis nearly 1000 years thenceforth, one might expect the networks to be less encumbered by the bias and partisanship that permeates more qualitative aspects of the text. In this sense, the networks approach delivers unique insights in that it extracts a perhaps unintended message from his time, namely new, quantitative knowledge of the Viking Age in Ireland.

## 6. Conclusions

The purpose of this paper is to gain quantitative insight into the complexity and conflicts of the Viking Age in Ireland as described in *Cogadh Gaedhel re Gallaibh*. A literal interpretation of ‘the popular tradition of Clontarf as wholly an Irish-Norse’ conflict [5] would suggest a strongly negative value of categorical assortativity for the negative (conflictual) network. On the other hand, the revisionist picture of a ‘civil war’ [2], an ‘internal struggle’ [6], with Leinster as the ‘predominant element’ [5] or ‘principal rebels’ [13], suggests a positive value of categorical assortativity for the negative network. The primary outcome of our investigation is our measured value of the associated metric and we find a negative value, supportive of the traditional picture. But its magnitude is moderate, suggesting that, at least in network terms, *Cogadh Gaedhel re Gallaibh* does not describe a fully ‘clear-cut’ Irish versus Norse conflict. The power of our analysis is that we can quantify this statement, and the value *ρ*=−0.32 means *Cogadh Gaedhel re Gallaibh* describes the Viking Age in Ireland as predominantly an Irish-Norse conflict, but it is not wholly so.

There are a number of other ways in which this work can be extended. Like [49,50], the present analysis is based on static networks. These freeze the narrative progress and capture the plot ‘all at one glance in a visual display of its character network’ [72]. Static networks are particularly advantageous for *Cogadh Gaedhel re Gallaibh* which, although believed to have been composed following some of the annals, paid limited regard to chronology [30,48]. Nonetheless, dynamical properties are also of interest and should be investigated in the future [61]. It would be interesting to see if temporal networks can help restore some of the chronology to *Cogadh Gaedhel re Gallaibh* [71]. Directed and weighted networks also offer obvious routes for wider study. Furthermore, motivated by the Ua Ruairc example, it would also be interesting to investigate if the structural imbalance in some network triads could be developed to give a way to spot other potential interpolations, not least because the survival of only one complete manuscript limits opportunities to identify interpolations through comparisons [48]. Another question is how the *Cogadh* narrative compares to others of the epic genre [49–51]. A comparison to the *Iliad* would be especially important as a link to an Irish account of the Trojan War (*Togail Troí*—‘The Destruction of Troy’) has been suggested before by humanities scholars, using traditional methods [19,23,29,42]. It would be interesting to continue such comparative investigations at a more detailed level in future studies.

A criticism sometimes levelled at the character-network approach is that it brings little new; merely confirming knowledge already gained from traditional approaches to humanities. The rebuttal to such criticism is that agreement is precisely what one would expect from a new approach which is valid and still evolving. The quantitative determination of categorical assortativity in this paper, and its precise placement of *Cogadh Gaedhel re Gallaibh* along the spectrum from the international to the intranational, is a new development in the evolution of this field. In that sense, our paper goes beyond limitations identified in some previous works in that it generates a new quantitative element to an unfinished debate in the humanities.

## Figures and Tables

**Figure 4.**
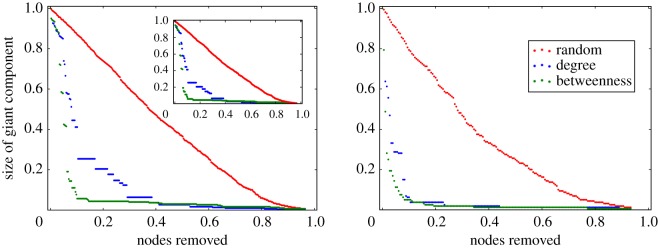
The relative sizes of the giant components as a function of the percentage of nodes removed. In the left panel the size of the giant component for the unsigned network is given. That of the positive network, which has a very similar decay, is given as an insert. The right panel shows the decay of the giant component of the negative network as nodes are removed. The red data points correspond to random removal of nodes and the blue and green data concern removal by highest degree and betweenness, respectively.

## Footnotes

1 Alternative spellings exist in the literature, but we employ the spelling used by James Henthorn Todd [4] since his is the edition that we analyse. We sometimes refer to the narrative simply as ‘the *Cogadh*’ hereafter.

2 There are a number of etymological theories for the word ‘Viking’ [47]. We use it to refer to the medieval Norse or Scandinavian raiders and invaders who attacked Ireland (and other countries) by sea, or those who subsequently settled in Ireland, between the late eighth and eleventh centuries [43]. A stricter definition of the term ‘Viking’ may involve the notion of ‘piratical’ and in this sense, not all Vikings were Scandinavian and not all Scandinavians were Vikings [43]. But we use the term in the looser sense (in keeping with much of the literature, e.g. [27,30,33,34,36,38,40,41]). These are the *Gaill* (singular *Gall*) referred to above.

3 We are aware that terms related to the word ‘national’ may be viewed as anachronistic here [13]; we use them in the sense of a large group of people with common characteristics such as language, traditions, customs and ethnicity [33], rather than in a governmental sense [56].

4 ‘Uí’ means ‘grandchildren’ or descendants so that the Uí Briain are the descendants of Brian and the Uí Néill are descendants of Niall, etc. ‘Ua’ is the singular form.

5 The term is motivated by a discussion in [33] of ‘the strong sense of identity, achievement, and cultural cohesion that had been created by the Irish learned classes’. Ó Corráin states ‘The island was united culturally and linguistically’ and ‘Self-consciously, the literati saw the Irish as a people or *natio*, to be compared with the Goths, the Franks, or the peoples of classical antiquity. As far as the genealogists were concerned, the Vikings were outsiders, and were called *Gaill* “Foreigners” to the end. Irish reaction to the Vikings is to be understood in terms of these cultural traits.’ For further discussions of Hiberno-Scandinavian relations, see, e.g. [34,47].

6 Ireland’s most powerful kings were described—either by themselves, or retrospectively—as king of Tara and less commonly, ardrí (translated as ‘high king’). These concepts were emphasized by the Uí Néill dynasty who claimed high kingship on the basis of their holding of Tara which long had a special status in Ireland’s polity. The kingship of Tara rotated between the northern and southern branches of the Uí Néill until Máel Sechnaill mac Domnaill’s claim to the title was interrupted by Brian Boru. However, assertions of high kingship were just that—claims rather than unopposed fact. The law tracts gave only three grades of king but no ‘high king’ or king of Ireland. For discussion of the nature of kingship and its various grades in Ireland, see, e.g. [6,7,59].

7 Actually, we identified 326 individual characters in total. Of these, 11 are isolated in the sense that they do not interact in the narrative. We consider these as not forming part of the *Cogadh* network and they are omitted from our analysis. The characters were identified in the main part of Todd’s text. Todd’s paratexts (introduction, footnotes, appendices and index) were used to aid the identification of characters and links between them but individuals mentioned only in the paratexts do not form part of the *Cogadh* network. A small number of characters appear in the main text but are omitted in Todd’s index. We also identified 34 groups of unnamed characters. If considered as nodes, they bring an additional 187 edges. However because these are neither individuals nor named, we omit them from our presentation too. Besides, and for completeness, we also analysed the networks with these nodes included and they deliver only very small changes to the statistics presented here.

8 Again we have omitted isolated nodes from the positive and negative sub-networks.

9 The error here is estimated using the method described in [65,66]. Error estimates for other network statistics are small (see discussion in the final paragraph of §3.3) and we refrain from reporting them here. We only display assortativity errors because they provide useful information when comparing systems which are, or nearly are, uncorrelated (*r* close to zero).

10 As usual, isolated (degree-zero) nodes are removed. For example, there are 202 Irish nodes in total (see table 3), but 9 of these are disconnected from other Irish nodes, so they are omitted from the unsigned Irish network in table 2. Besides the value of *N*, reinstating them does not alter the statistics listed within the precision of table 2.

11 Some of the entries in the second and third rows of table 3 differ from entries in the third column of table 2 because isolated nodes are not removed from sub-networks in table 3. This is because table 3 concerns identity profiles of unsigned, positive and negative networks, in distinction to the Irish and Viking sub-networks of table 2. Numbers of edges match across both tables, however, because, by definition, these do not involve isolated nodes.

## Data accessibility

Data are available from https://github.com/ralphkenna/CGG.

## Appendix A. Ireland during the Viking Age

The five provinces referred to in the main text are Connacht, Leinster, Ulster, Meath and Munster. Their names are associated, respectively, with member populations called the Connachta, the Laigin, the Ulaid, and the kingdoms of Mide and of Mumu. The modern province of Ulster encompasses the territories of the Northern Uí Neill and Ulaid (from which Ulster derives its name), as well as parts of Bréifne and Airgíalla. Mide, associated with the Southern Uí Néill, mainly comprised the modern county Westmeath and part of Meath and has been subsumed into the modern Leinster. In the tenth century the main rivalry for claims to high kingship of Ireland was between the northern and southern branches of the Uí Néill. Their dominance was ended by Brian Boru.

In the ninth century, Cork, Dublin, Limerick, Waterford and Wexford all developed from Viking base camps to more permanent settlements. See figure 3 which, alongside an image adopted from the Book of Leinster for Todd’s edition of *Cogadh Gaedhel re Gallaibh*, includes a map outlining the political structure of Ireland about AD 900.

## Appendix B. Scalar and categorical assortativity

In the main text, we used two different forms of assortativity: the degree assortativity *r* and the measure *ρ*. The first of these is an example of *scalar assortativity*—it quantifies the tendency of nodes whose degrees have similar values to associate with each other. In determining *r*, it is important to account for nodes possibly having similar but not identical values; e.g. high degree nodes may tend to mix with other high degree nodes without them having to have precisely the same *k*-values. The second is *categorical*—it measures tendencies for nodes belonging to the same category to link to each other. In the categorical case, two nodes either have the same attributes or they do not; there is no question of degrees of similarity here. Therefore, we require two different formulae to quantify scalar and categorical assortativity.

Scalar assortativity is simply given by Pearson’s correlation coefficient, i.e. it is the covariance of two variables normalized by the product of their standard deviations. The normalization factor ensures that the assortativity takes values in the range [−1,1]. Networks with a degree value *r*>0 are termed *degree assortative*. If the measured value of *r* is negative, the network is deemed *degree disassortative*. Since the theoretical bounds on scalar assortativity are the same for all networks, comparisons of assortativity between them are straightforward and meaningful.

Many networks tend to evolve towards their maximum-entropy state unless otherwise constrained [73]. Such maximum-entropy states are usually disassortative because disassortative configurations are more abundant than assortative ones [74]. For this reason, non-social networks are usually degree-disassortative. Social networks, on the other hand, are usually uncorrelated or assortative. This can be explained by homophily; highly connected people tend to link together [65,66]. The lack of disassortativity in the positive networks, as seen in table 2, is a common feature of epic narratives. It is a signal of the presence of a non-trivial social or narrative force—driving them away from their maximum-entropy, anticorrelated (disassortative) states. In this sense, positive character networks, including those of *Cogadh Gaedhel re Gallaibh* are more like social networks than unlike them.

For categorical assortativity, consider the nodes *i* of a network having attributes *c*_*i*_ which could be colours (e.g. green, blue or grey) as in the main text. We require the difference between the fraction of edges that exist between nodes of the same attribute and the fraction of such edges we would expect if the nodes were connected at random regardless of the nodes’ attributes (i.e. if the linking process were ‘colour blind’). It is defined as follows [65,66].

The total degree of the network is $\sum_{i=1}^{N}k_{i}=2M$ (twice the number of edges because each edge is double counted). Let *c* and *c*^′^ denote categorical variables and let *e*_*cc*^′^_ denote the density of directed edges in the network pointing from nodes of type *c* to nodes of type *c*^′^. We note that *e*_*cc*^′^_=*e*_*c*^′^*c*_ if the network is undirected. We define the density of degrees associated with nodes of type *c* as

B 1 $$a_{c}=\sum_{c^{\prime }}e_{cc^{\prime }}=\frac{1}{2M}\sum_{i}k_{i}\delta_{c_{i}c},$$

and have the sum rule

B 2 $$\sum_{c}a_{c}=\sum_{cc^{\prime }}e_{cc^{\prime }}=1.$$

The *modularity* is defined as

B 3 $$Q=\sum_{c}\left(e_{cc}-a_{c}^{2}\right).$$

The categorical assortativity *ρ* is obtained by normalizing the modularity so that its maximum value is 1 (as is the case for the scalar assortativity). If the network is fully assortative, *all* edges connect nodes of the same type. Therefore, the normalizing factor for *Q* is given by equation (B 3) with $\sum_{c}e_{cc}$ set to 1. This motivates the definition

B 4 $$\rho =\frac{\sum_{c}\left(e_{cc}-a_{c}^{2}\right)}{1-\sum_{c}a_{c}^{2}}.$$

The minimum possible value of this quantity is obtained when all edges connect nodes of different types (*e*_*cc*_=0 for all *c*) and is

B 5 $$\rho_{\min}=\frac{-\sum_{c}a_{c}^{2}}{1-\sum_{c}a_{c}^{2}}.$$

Fully disassortative, undirected networks with only two categories have *ρ*_min_=−1. However, the minimum value for *ρ* is not generally −1 if more categories are involved. While the absence of assortativity means that $\sum_{c}e_{cc}=0$ for any number of categories, the lack of directedness that assures the symmetry between the categories only happens when there are two of them. This property, together with equation (2) trivially gives *ρ*=−1. More generally, *ρ*_min_ lies between −1 and 1.

The reason why *ρ*_min_ is not −1 is for a perfectly disassortative network is that such a network more closely resembles a random network than does a perfectly assortative one when there are more than two categories, i.e. random mixing mostly mixes unlike nodes and disassortativity does the same. But assortativity mixes like nodes. This is why the minimum value of *ρ* is closer to the value for a random network *ρ*=0 than is the maximum value *ρ*=1. In the main text, we have to be mindful of this when interpreting the categorical assortativity for the negative network. However, we could easily introduce a measure which is −1 for a fully disassortative network as follows.

The modularity in equation (B 4) is defined with respect to the expected density of edges between nodes of the *same* category if the network were assembled without regard to category. This was appropriate for the measurement of *assortativity*. To directly measure *disassortativity* instead, we focus on edges between nodes of *different* categories and introduce

B 6 $$\bar{\rho }=-\frac{\sum_{c,c^{\prime }}^{\prime }\left(e_{cc^{\prime }}-a_{c}a_{c^{\prime }}\right)}{1-\sum_{c,c^{\prime }}^{\prime }a_{c}a_{c^{\prime }}},$$

where the prime on the summation means that it is taken over *c* and *c*^′^ values such that *c*≠*c*^′^ and the leading minus sign is to ensure that disassortative networks have negative $\bar{\rho }$-values, in line with their negative *ρ*-values.

Equation (B 2) gives

$$\sum_{c,c^{\prime }}^{\prime }e_{cc^{\prime }}=1-\sum_{c}e_{cc},$$

enabling us to write

B 7 $$\bar{\rho }=\rho \left(\frac{1}{\sum_{c}a_{c}^{2}}-1\right).$$

From equation (B 5), this may be written

B 8 $$\bar{\rho }=-\frac{\rho }{\rho_{\min}}.$$

In other words, $\bar{\rho }$ is simply the assortativity normalized by its minimum possible value (which is negative). This has the advantage that its value is 1 for a fully disassortative network; however, a fully assortative network may have a value of $\bar{\rho }$ which exceeds 1.

We therefore introduce a renormalized version of the categorical assortativity that is suitable for all circumstances

B 9 $$\hat{\rho }=\left\{ \begin{array}{ll} \rho & \text{if }\rho >0,\\ -\frac{\rho }{\rho_{\min}} & \text{if }\rho <0. \end{array}\right.$$

This measure has the desired features that it vanishes in the case of colour blindness, and it is 1 and −1 for fully assortative and fully disassortative networks, respectively. In table 5, we list the values of $\hat{\rho }$ for the various networks. This may be considered as a renormalized version of table 4 of the main text. The differences between the values entered in the two tables are very small.

## Appendix C. Network robustness and importance of individual characters

Having investigated the giant component in the main text, we may ask how reliant its integrity is on the most important characters. This is a question of robustness and one investigates it by determining the effects of systematic and random removal of nodes or edges. In the former approach, we remove the most important nodes one by one and monitor how the giant component reduces in size. We can then compare this to the results of the latter approach, in which removal of nodes is a random process.

There are a number of ways in which we can decide which are the most important or influential nodes. One way is to consider that those with highest degree are most important and to remove them first. Another possibility is to consider nodes with the highest *betweenness centralities* [75]. This counts the number of shortest paths (geodesics) which pass through each node [75]. To define it, we first write the number of geodesics between nodes *i* and *j* as *σ*(*i*,*j*). We denote the number of these which pass through node *l* as *σ*_*l*_(*i*,*j*). The betweenness centrality of vertex *l* is then defined as

C 1 $$g_{l}=\frac{2}{\left(N-1)(N-2\right)}\sum_{i\neq j}\frac{\sigma_{l}(i,j)}{\sigma (i,j)}.$$

If *g*_*l*_=1, all geodesics pass through node *l*. If *i*,*j* and *l* represent edges rather than nodes, equation (C 1) can be interpreted as the *edge betweenness centrality* instead.

Other measures of importance include nodes’ *closeness* and *eigenvector centralities*. The sum of the distances of a given node from all other nodes in a connected graph or component is termed its *farness*. The reciprocal of farness is a measure of how central a node is and is termed its *closeness* [54]. *Eigenvector centrality* characterizes node importance in terms of centralities of its neighbours; nodes are deemed influential according to how they are linked to other important nodes [54]. Eigenvector centrality is a variant of the ‘pagerank’ score used to rank websites. The leading characters of *Cogadh Gaedhel re Gallaibh* are listed in table 6, ranked according to four different measures: degree; betweenness; closeness and eigenvector centrality.

**Table 6.** The most important characters of *Cogadh Gaedhel re Gallaibh* ranked according to their degree, betweenness centrality, closeness and eigenvector centrality.

rank	degree	betweenness	closeness	eigenvector
unsigned				
1	Brian (105)	Brian (0.42)	Brian (0.44)	Brian (0.53)
2	Sitriuc (62)	Sitriuc (0.21)	Sitriuc (0.41)	Maelmordha (0.28)
3	Maelmordha (42)	Ottir (0.16)	Ottir (0.39)	Máel Sechnaill (0.22)
4	Ottir (40)	Aedh Finnliath (0.13)	Gormflaith (0.38)	Sitriuc (0.21)
5	Máel Sechnaill (36)	Ossill (0.11)	Maelmordha (0.38)	Gormflaith (0.21)
positive				
1	Brian (53)	Brian (0.28)	Sitriuc (0.34)	Brian (0.48)
2	Sitriuc (40)	Sitriuc (0.17)	Brian (0.34)	Murchadh (0.30)
3	Maelmordha (38)	Máel Sechnaill (0.11)	Gormflaith (0.34)	Maelmordha (0.26)
4	Gormflaith (34)	Ottir (0.10)	Maelmordha (0.32)	Máel Sechnaill (0.26)
5	Ottir (32)	Gormflaith (0.10)	Máel Sechnaill (0.32)	Conaing (0.23)
negative				
1	Brian (63)	Brian (0.63)	Brian (0.44)	Brian (0.66)
2	Sitriuc (25)	Ottir (0.23)	Máel Sechnaill (0.35)	Maelmordha (0.23)
3	Mathgamhain (17)	Sitriuc (0.23)	Sitriuc (0.34)	Brodar (Brodir) (0.22)
4	Cathal (14)	Aedh Finnliath (0.16)	Ottir (0.33)	Máel Sechnaill (0.17)
5	Olaf Cuaran (12)	Olaf Cuaran (0.12)	Ivar (0.32)	Ivar (0.17)

We present the study of robustness for the networks underlying *Cogadh Gaedhel re Gallaibh* in figure 4. The main left panel depicts the relative sizes of the giant component of the unsigned network as nodes are removed randomly (red data points), by highest betweenness (blue) and by degree (green). A similar behaviour is observed for the positive network, shown in the insert. The counterpart information for the negative sub-network is contained in the next panel. We see that random removal of nodes only has a relatively gradual effect on the giant-component size in all three networks. Removal by betweenness or by degree has far more devastating consequences. Removal by betweenness is particularly damaging for the integrity of the full and positive networks, whereas, for the negative network, removal by betweenness and degree are about equally effective. Details of the effects of node-removal on the relative sizes of the giant components are given in table 7.

**Table 7.** The effects of removing the most important characters or of removing characters at random. The entries in the table give the relative size of the giant component after removal of the top 10% of characters systematically and randomly; the top five characters; and after removal of the most important character, namely Brian Boru.

	remove 10% by degree	remove 10% by betweenness	remove 10% randomly	remove top 5 by degree	remove top 5 by betweenness	remove Brian Boru
unsigned	43%	6%	92%	90%	91%	92%
positive	47%	7%	83%	85%	85%	86%
negative	6%	5%	81%	69%	58%	85%

## References

[1] Kenna R, MacCarron M, MacCarron P 2017 *Maths meets myths: quantitative approaches to ancient narratives*. Berlin, Germany: Springer International Publishing.

[2] O’Connor C 1766 *Dissertations on the History of Ireland. To which is subjoined, a dissertation on the Irish colonies established in Britain. With some remarks on Mr. Mac Pherson’s translation of Fingal and Temora*, 2nd edn Dublin, Ireland: G. Faulkner.

[3] O’Halloran S 1778 *A general History of Ireland, from the earliest accounts to the close of the twelfth century, collected from the most authentic records*, vol. III London, UK: A. Hamilton.

[4] Todd JH (ed. and trans.) 1867 *Cogadh Gaedhel re Gallaibh. The war of the Gaedhil with the Gaill, or, the invasions of Ireland by the Danes and other Norsemen*. London, UK: Longmans, Green, Reader, and Dyer.

[5] Ryan J 1938 The battle of Clontarf. *J. R. Soc. Antiq. Ireland* 8, 1–50.

[6] Ó Corráin D 1972 *Ireland before the Normans*. Dublin, Ireland: Gill and MacMillan.

[7] Duffy S 2014 *Brian Boru and the battle of Clontarf*. Dublin, Ireland: Gill and Macmillan Ltd.

[8] Birkett T, Lee C (eds) 2014 *The Vikings in Munster*. Nottingham, UK: Centre for the Study of the Viking Age, University of Nottingham.

[9] Casey D, Meehan B 2014 Brian Boru and the book of Armagh. *Hist. Ireland* 22, 28–29.

[10] Clarke HB, Johnson R (eds) 2015 *Before and after the battle of Clontarf: the Vikings in Ireland and beyond*. Dublin, Ireland: Four Courts Press.

[11] Duffy S (ed.). 2017 Medieval Dublin XVI. In *Proc. Clontarf 1014–2014: Natl Conf. Marking the Millennium of the Battle of Clontarf, Dublin, 11–12 April 2014*. Dublin, Ireland: Four Courts Press.

[12] Duffy S 2014 Brian Boru at the Battle of Clontarf: a medieval version of 1916? *Irish Times*, 25 January 2014. See http://www.irishtimes.com/culture/heritage/brian-boru-at-the-battle-of-clontarf-a-medieval-version-of-1916-1.1667155 (accessed 2 December 2017).

[13] Etchingham C 2015 His finest hour. *Irish Lit. Suppl.* 34, 3–4. made available through The Free Library (22 March 2015). https://www.thefreelibrary.com/Hisfinesthour.-a0407227269 (accessed 2 December 2017).

[14] McGettigan D 2013 *The battle of Clontarf, Good Friday, 1014*. Dublin, Ireland: Four Courts Press.

[15] Royal Irish Academy. 2014 Humanities: 1014 Battle of Clontarf—Royal Irish Academy Lunchtime Lecture Series Spring 2014. See https://soundcloud.com/the-royal-irish-academy/sets/royal-irish-academy-lunchtime-lecture-series-spring-2014-1014 (accessed 2 December 2017).

[16] 1014: Brian Boru and the Battle of Clontarf—History Hub. See http://historyhub.ie/1014-brian-boru-battle-of-clontarf (accessed 2 December 2017).

[17] Ireland’s Troy? See http://www.cam.ac.uk/research/news/irelands-troy (accessed 2 December 2017).

[18] Downham C 2014 Clontarf in the wider world. *Hist. Ireland* 22, 22–29.

[19] Bugge A (ed. and trans.). 1905 *Caithreim Chellachain Chaisil: the victorious career of Cellachan of Cashel or the wars between the Irishmen and the Norsemen in the middle of the 10th century*. Oslo, Norway: Gundersen.

[20] Bugge S 1908 *Norsk sagafortælling og sagaskrivning i Irland*. Oslo, Norway: Grøndahl.

[21] Meyer K 1910 Brian Borumha. *Ériu* 4, 68–73.

[22] Thurneysen R 1921 *Die irische Helden- und Königsage bis zum siebzehnten Jahrhundert*. Halle, Germany: Max Niemeyer.

[23] Goedheer AJ 1938 *Irish and Norse traditions about the battle of Clontarf*. Haarlem, The Netherlands: H.D. Tjeenk, Willink & Zoon.

[24] Ryan J 1940 Review of ‘Irish and Norse traditions about the Battle of Clontarf’ by Albertus Johannes Goedheer. *Ir. Hist. Stud.* 2, 93–97.

[25] Flower R 1947 *The Irish tradition*. Oxford, UK: Oxford University Press.

[26] Nic Ghiollamhaith A 1981 Dynastic warfare and historical writing in North Munster, 1276–1350. *Cambridge Mediev. Celtic Stud.* 2, 73–89.

[27] Ní Mhaonaigh M 1992 Bréifne bias in Cogad Gáedel Re Gallaib. *Ériu* 43, 135–58.

[28] Holm P 1994 Between apathy and antipathy: the Vikings in Irish and Scandinavian history. *Peritia* 8, 151–169. (doi:10.1484/J.Peri.3.209)

[29] Ní Mhaonaigh M 1995 Cogad Gáedel re Gallaib: some dating considerations. *Peritia* 9, 354–377. (doi:10.1484/J.Peri.3.255)

[30] Ní Mhaonaigh M 1996 Cogad Gáedel re Gallaib and the annals: a comparison. *Ériu* 47, 101–126.

[31] Ní Mhaonaigh M 1997 Some Middle Irish declensional patterns in Cogad Gáedel re Gallaib. *Z. Celt. Philol.* 49, 615–628. (doi:10.1515/zcph.1997.49-50.1.615)

[32] Ó Corráin D 1997 Ireland, Wales, and the Hebrides. In *The Oxford illustrated history of the Vikings* (ed. P Sawyer), pp. 105–106. Oxford, UK: Oxford University Press.

[33] Ó Corráin D 1998 Viking Ireland: afterthoughts. In *Ireland and Scandinavia in the early Viking Age* (eds HB Clarke, M Ní Mhaonaigh, R Ó Floinn), pp. 421–452 Dublin, Ireland: Four Courts Press.

[34] Ní Mhaonaigh M 1998 Friend and foe: Vikings in ninth- and tenth-century Irish literature. In *Ireland and Scandinavia in the early Viking Age* (eds HB Clarke, M Ní Mhaonaigh, R Ó Floinn) pp. 381–402. Dublin, Ireland: Four Courts Press.

[35] Ní Mhaonaigh M 2002 Tales of three Gormlaiths in medieval Irish literature. *Ériu* 52, 1–24.

[36] Downham C 2005 The battle of Clontarf in Irish history and legend. *Hist. Irel.* 13, 19–23.

[37] Casey D 2010 Historical and literary representations of Brian Boru’s burial in Armagh, 1014 AD. *North Munster Antiquarian J.* 50, 29–44.

[38] Dubois TA 2011 Juxtaposing Cogadh Gáedel re Gallaib with Orkneyinga saga. *Oral Tradition* 26, 267–296. (doi:10.1353/ort.2011.0029)

[39] Ní Úrdail M 2011 *Cath Cluana Tarbh ‘The battle of Clontarf’*. London, UK: The Irish Texts Society/Cumann na Scríbheann nGaedhilge.

[40] Ní Mhaonaigh M 2012 A neglected account of the battle of Clontarf. *Z. Celt. Philol.* 59, 143–167.

[41] Casey D 2013 A reconsideration of the authorship and transmission of Cogadh Gáedhel re Gallaibh. *Proc. R. Irish Acad. C* 113, 139–161. (doi:10.3318/PRIAC.2013.113.03)

[42] Ní Mhaonaigh M 2014 ‘The metaphorical Hector’: the literary portrayal of Murchad mac Bríain. In *Classical literature and learning in medieval Irish narrative* (ed. R O’ Connor), pp. 140–161. Cambridge, UK: Boydell and Brewer.

[43] Kirwan M 2014 The Viking legacy in Ireland. In *The Vikings in Munster* (eds T Birkett, C Lee), pp. 4–6. Nottingham, UK: Centre for the Study of the Viking Age, University of Nottingham.

[44] Smyth AP 1974–1977 The black foreigners of York and the white foreigners of Dublin. In *Saga-book of the Viking society*, vol. 19 (ed. AR Taylor), pp. 101–117. London, UK: Viking Society for Northern Research, University College London.

[45] Downham C 2007 *Viking Kings of Britain and Ireland: the dynasty of Ívarr to A.D. 1014*. Edinburgh, UK: Dunedin Academic Press.

[46] Downham C 2012 Scottish affairs and the political context of Cogadh Gaedhel re Gallaibh. In *Traversing the inner seas: contacts and continuity around Western Scotland, the Hebrides and Northern Ireland* (ed C. Cooijmans), pp. 86–106. Edinburgh, UK: Scottish Society for Northern Studies.

[47] Downham C 2012 Viking ethnicities: a historiographic overview. *Hist. Compass* 10, 1–12. (doi:10.1111/j.1478-0542.2011.00820.x)

[48] Downham C 2012 The ‘annalistic section’ of Cogad Gáedel re Gallaib. *Peritia* 24–25, 141–172. (doi:10.1484/J.PERIT.5.102744)

[49] Mac Carron P, Kenna R 2012 Universal properties of mythological networks. *Europhys. Lett.* 99, 28002 (doi:10.1209/0295-5075/99/28002)

[50] Mac Carron P, Kenna R 2013 Network analysis of the Íslendinga sögur—the Sagas of Icelanders. *Eur. Phys. J. B* 86, 407 (doi:10.1140/epjb/e2013-40583-3)

[51] Yose J, Kenna R, Mac Carron P, Platini T, Tonra J 2016 A networks-science investigation into the epic poems of Ossian. *Advs. Complex Syst.* 19, 1650008 (doi:10.1142/S0219525916500089)

[52] Albert R, Barabási AL 2002 Statistical mechanics of complex networks. *Rev. Mod. Phys.* 74, 47–94. (doi:10.1103/RevModPhys.74.47)

[53] Newman MEJ 2003 The structure and function of complex networks. *SIAM Rev.* 45, 167–256. (doi:10.1137/S003614450342480)

[54] Newman MEJ 2010 *Networks: an introduction*. Oxford, UK: Oxford University Press.

[55] Costa LF, Oliveira ON Jr, Travieso G, Rodrigues FA, Villas Boas PR, Antiqueira L, Viana MP, Da Rocha LEC 2011 Analyzing and modeling real-world phenomena with complex networks: survey of applications. *Adv. Phys.* 60, 329–412. (doi:10.1080/00018732.2011.572452)

[56] Nation. 2017 See https://en.wikipedia.org/wiki/Nation (accessed 2 December 2017).

[57] Harrison S 2014 The battle of Clontarf: the archaeological evidence? Talk as part of the Royal Irish Academy Lunchtime Lecture Series, Spring 2014. See https://soundcloud.com/the-royal-irish-academy/stephen-harrison (accessed 2 December 2017).

[58] Sayers W 1991 Clontarf, and the Irish destinies of Sigurðr Digri, Earl of Orkney, and Þorsteinn Síðu-Hallsson. *Scand. Stud.* 63, 164–186.

[59] Byrne FJ 1973 *Irish kings and high-kings*. London, UK: B.T. Batsford Ltd.

[60] Mac Carron P 2014 A network theoretic approach to comparative mythology. PhD thesis, Coventry University, Coventry, UK.

[61] Prado SD, Dahmen SR, Bazzan AL, MacCarron P, Kenna R 2016 Temporal network analysis of literary texts. *Adv. Complex Syst.* 19, 1650005 (doi:10.1142/S0219525916500053)

[62] Trovati M, Brady J 2014 Towards an automated approach to extract and compare fictional networks: an initial evaluation. In *Proc.—Int. Workshop on Database and Expert Systems Applications*, *DEXA*, *Munich, Germany, 1–5 September*, pp. 246–250. IEEE (doi:10.1109/DEXA.2014.58)

[63] Moretti F 2011 Network theory, plot analysis. *New Left Review* 68, 80–102.

[64] McPherson M, Smith-Lovin L, Cook JM 2001 Birds of a feather: homophily in social networks. *Annu. Rev. Sociol.* 27, 415–444. (doi:10.1146/annurev.soc.27.1.415)

[65] Newman MEJ 2002 Assortative mixing in networks. *Phys. Rev. Lett.* 89, 208701 (doi:10.1103/PhysRevLett.89.208701) 12443515 10.1103/PhysRevLett.89.208701

[66] Newman MEJ 2003 Mixing patterns in networks. *Phys. Rev. E* 67, 026126 (doi:10.1103/PhysRevE.67.026126) 10.1103/PhysRevE.67.026126 12636767

[67] Newman MEJ, Park J 2003 Why social networks are different from other types of networks. *Phys. Rev. E* 68, 036122 (doi:10.1103/PhysRevE.68.036122) 10.1103/PhysRevE.68.036122 14524847

[68] Heider F 1946 Attitudes and cognitive organization. *J. Psychol.* 21, 107–112. (doi:10.1080/00223980.1946.9917275) 21010780 10.1080/00223980.1946.9917275

[69] Cartwright D, Harary F 1956 Structural balance: a generalization of Heider’s theory. *Psychol. Rev.* 63, 277–293. (doi:10.1037/h0046049) 13359597 10.1037/h0046049

[70] Antal T, Krapivsky PL, Redner S 2006 Social balance on networks: the dynamics of friendship and enmity. *Phys. D* 224, 130–136. (doi:10.1016/j.physd.2006.09.028)

[71] Mc Carthy DP 2008 *The Irish annals—their genesis, evolution and history*. Dublin, Ireland: Four Courts Press.

[72] Rhody LM 2011 A method to the model: responding to Franco Moretti’s network theory, plot analysis. See https://magmods.wordpress.com/2011/08/22/a-method-to-the-model-responding-to-franco-moretti (accessed 3 July 2017).

[73] Bianconi G 2007 The entropy of randomized network ensembles. *Europhys. Lett.* 81, 28005 (doi:10.1209/0295-5075/81/28005)

[74] Johnson S, Torres JJ, Marro J, Muñoz MA 2010 The entropic origin of disassortativity in complex networks. *Phys. Rev. Lett.* 104, 108702 (doi:10.1103/PhysRevLett.104.108702) 20366458 10.1103/PhysRevLett.104.108702

[75] Freeman LC 1977 A set of measures of centrality based on betweenness. *Sociometry* 40, 35–41. (doi:10.2307/3033543)

